# Pathogenic Roles of Heparan Sulfate and Its Use as a Biomarker in Mucopolysaccharidoses

**DOI:** 10.3390/ijms231911724

**Published:** 2022-10-03

**Authors:** Kohtaro Minami, Hideto Morimoto, Hiroki Morioka, Atsushi Imakiire, Masafumi Kinoshita, Ryuji Yamamoto, Tohru Hirato, Hiroyuki Sonoda

**Affiliations:** Research Division, JCR Pharmaceuticals, Kobe 651-2241, Japan

**Keywords:** heparan sulfate, mucopolysaccharidoses, lysosomal storage disorders, cerebrospinal fluid, biomarker, blood-brain barrier

## Abstract

Heparan sulfate (HS) is an essential glycosaminoglycan (GAG) as a component of proteoglycans, which are present on the cell surface and in the extracellular matrix. HS-containing proteoglycans not only function as structural constituents of the basal lamina but also play versatile roles in various physiological processes, including cell signaling and organ development. Thus, inherited mutations of genes associated with the biosynthesis or degradation of HS can cause various diseases, particularly those involving the bones and central nervous system (CNS). Mucopolysaccharidoses (MPSs) are a group of lysosomal storage disorders involving GAG accumulation throughout the body caused by a deficiency of GAG-degrading enzymes. GAGs are stored differently in different types of MPSs. Particularly, HS deposition is observed in patients with MPS types I, II, III, and VII, all which involve progressive neuropathy with multiple CNS system symptoms. While therapies are available for certain symptoms in some types of MPSs, significant unmet medical needs remain, such as neurocognitive impairment. This review presents recent knowledge on the pathophysiological roles of HS focusing on the pathogenesis of MPSs. We also discuss the possible use and significance of HS as a biomarker for disease severity and therapeutic response in MPSs.

## 1. Introduction

Heparan sulfate (HS), a glycosaminoglycan (GAG), is a linear sulfated polysaccharide consisting of a simple structure of repeating disaccharides, namely, *N*-acetylglucosamine (GlcNAc) and glucuronic acid or iduronic acid [1,2,3]. HS functions as a component of proteoglycans (heparan sulfate proteoglycans, HSPGs) on the cell surface and in the extracellular matrix of the basement membrane [1,2,3]. Based on their different locations and molecular features, HSPGs are classified into three groups: syndecans, having a single transmembrane domain; glypicans, anchored by glycosylphosphatidylinositol (GPI) to the plasma membrane, and secreted proteoglycans, including perlecan, agrin, and collagen XVIII [1,2,3,4].

**Figure 1:**
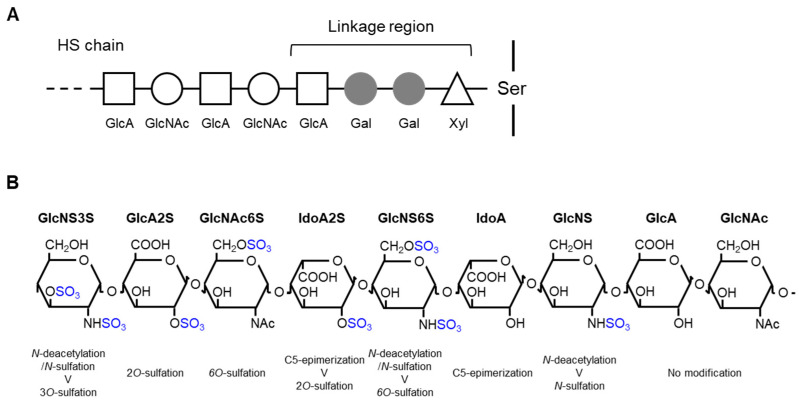
Schematic representation of heparan sulfate (HS) biosynthesis and modification: (**A**) HS biosynthesis, and (**B**) modification of the HS chain. The chain sequence is arbitrary to show possible modifications. Xyl, xylose; Gal, galactose; GlcA, glucuronic acid; GlcNAc, *N*-acetylglucosamine; Ser, serine; GlcNS, *N*-sulfated glucosamine; IdoA, iduronic acid.

The biosynthesis of HS, which occurs in the endoplasmic reticulum and Golgi apparatus, is initiated by the *O*-linked attachment of xylose to serine residues of core proteins followed by the linkage of two galactose residues and glucuronic acid (tetrasaccharide linkage region) to extend the repetitive disaccharide chain (Figure 1A). The chain elongation reaction begins with the linking of GlcNAc to the tetrasaccharide linkage region by the EXTL2/EXTL3 enzyme. Then, glycosyltransferase EXT1/EXT2 sequentially links the disaccharide chain. During these processes, the polysaccharide chain undergoes concurrent modifications, such as *N*-deacetylation/*N*-sulfation, C5-epimerization, and *O*-sulfation, which results in the structural diversity of HS chains (Figure 1B) [2,3,5,6].

HSPGs are involved in several physiological processes, including cell signaling, cell adhesion and motility, signaling molecule stabilization and localization in the extracellular matrix, and membrane trafficking [2]. These functions of HSPGs are largely attributed to their HS chains, although the core proteins themselves may affect the biological events [7,8,9]. The consequences of the genetic ablation of enzymes involved in HS metabolism have been investigated using gene-modified mouse models to reveal the critical roles of HSPGs, especially HS glycans, in many physiological processes [4,6]. Mutations in genes that encode the enzyme proteins associated with HS synthesis can cause human diseases. For example, deficiencies in *XYLT1* and *XYLT2*, both which encode xylosyl transferases and are involved in the linker synthesis, cause Desbuquois dysplasia type 2 and spondyloocular syndrome, respectively. Mutations in the genes encoding galactosyltransferases (*B4GALT7* and *B3GALT6*) cause spondylodysplastic types of Ehlers–Danlos syndrome [10,11]. Loss-of-function mutations in *EXT1*/*EXT2*, which encode exostosin glycosyltransferases, can lead to hereditary multiple exostoses [5,12].

**Figure 2:**
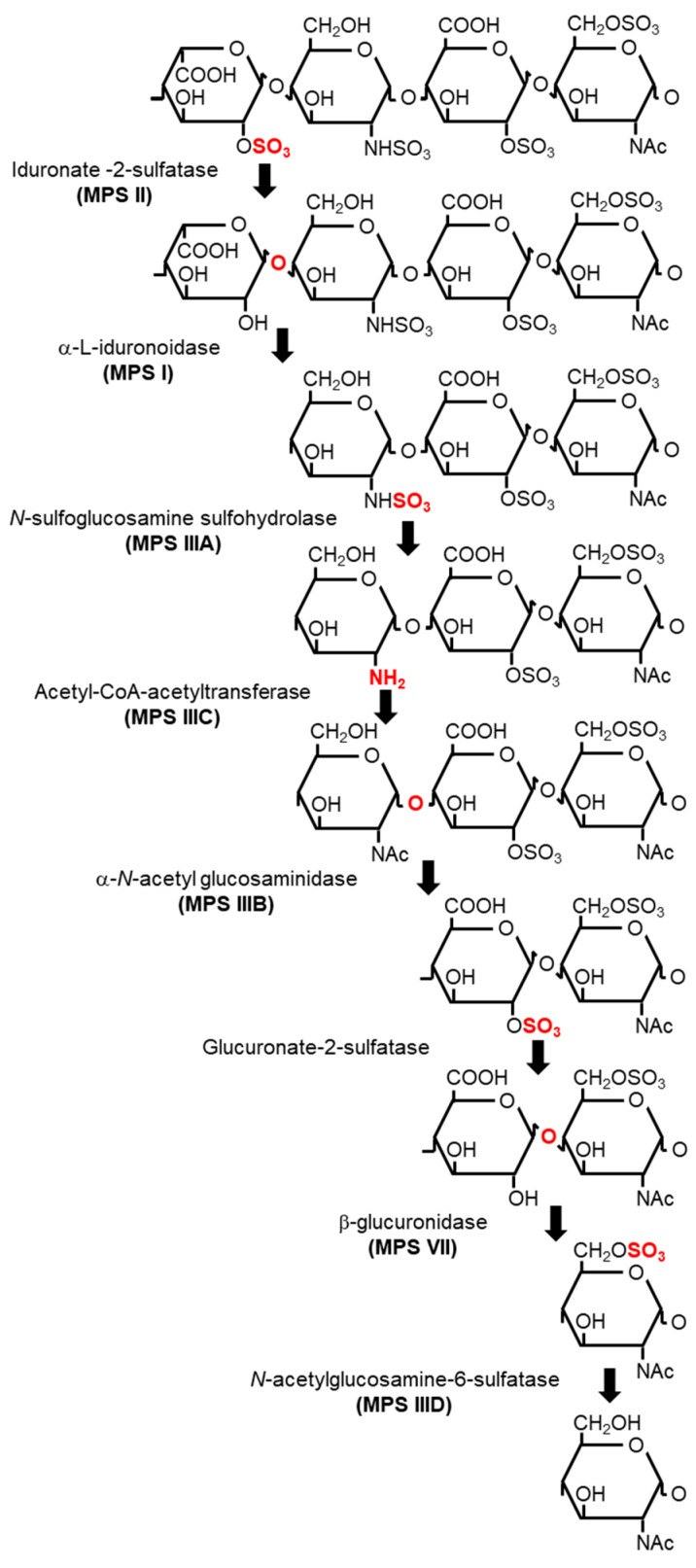
Sequential degradation of heparan sulfate (HS) and the catalyzing enzyme in each reaction. The type of mucopolysaccharidosis (MPS) caused by genetic defects is indicated in parentheses under the corresponding enzyme.

In addition to the biosynthesis, degradation of HS is an important biological process, which is largely mediated by a series of lysosome-resident enzymes (Figure 2). First, iduronate-2-sulfatase (IDS) catalyzes the removal of C2 sulfate from iduronate-2-sulfate (IdoA2S) at the non-reducing end of HS [13]. Second, α-L-iduronidase (IDUA) hydrolyzes the terminal α-L-iduronic acid (IdoA) residues of HS [14,15]. Third, *N*-sulfoglucosamine sulfohydrolase (SGSH) catalyzes the removal of sulfate from the amino group of the *N*-sulfated glucosamine residues of the HS chain [16,17]. Fourth, acetyl-CoA-acetyltransferase (HGSNAT) facilitates the acetylation of the terminal glucosamine residues of HS after de-sulfation by SGSH [18]. α-*N*-acetylglucosaminidase (NAGLU) cleaves the terminal GlcNac residues from the HS chain [19,20]. Glucuronate-2-sulfatase removes C2 sulfate from the 2-*O*-sulfated glucuronic acid residues of HS [21]. β-glucuronidase (GUSB) hydrolyzes the terminal b-D-glucuronic acid residues of HS [22]. *N*-acetylglucosamine-6-sulfatase (GNS) removes C6 sulfate from 6-O-sulfated GlcNac and the glucosamine residues of HS [23,24,25].

Defects in HS degradation can also cause human diseases. Since the degradation of proteoglycans occurs in lysosomes, the diseases are generally characterized by the accumulation of undigested GAGs in the lysosomes throughout the body, which are called mucopolysaccharidoses (MPSs) [26]. MPSs are a group of rare inherited metabolic disorders caused by the genetic mutations of lysosomal enzymes involved in GAG degradation. HS deposition is observed in MPSs I, II, III, and VII [27,28,29,30,31,32,33,34,35,36,37]. The causative gene mutation for each MPS has been identified (Figure 2).

This review presents recent knowledge on the physiological and pathophysiological roles of HS, especially focusing on the pathogenesis of MPSs. It also discusses the use and significance of HS as a biomarker for disease severity and therapeutic response in MPSs.

## 2. Physiological Roles of HS

HS presents and acts as a part of proteoglycans (HSPGs), namely, the transmembrane syndecans, the GPI-anchored glypicans, and secreted types of agrin, perlecan, and collagen XVIII [1,2,3,4]. The well-known roles of HSPGs include the regulation and modification of cell signaling as co-receptors. Cell-surface HSPGs are required for the formation of the FGF2-FGF receptor complex and for signal transduction [38,39]. HSPGs are involved in Wnt signaling and promote the interaction between Wnt and Frizzled receptors [40,41]. The Hedgehog, BMP, and VEGF signaling pathways are also regulated by HSPGs [42,43,44,45,46]. In addition to functioning as signaling co-receptors, HSPGs regulate morphogen gradient and movement [47,48]. Binding to HSPGs restricts the diffusion of morphogens along the cell surface to prevent extraordinal signal input to the cell in other layers [43]. Moreover, cell adhesion matrix proteins, such as fibronectin, vitronectin, and laminins, bind to cell surface HSPGs through their heparin-binding domains during cell adhesion and cell shape determination [49]. Accordingly, HS and HSPGs play critical roles in the morphogenesis of developing tissues and in the maintenance of normal cell functions of the body and intercellular communication throughout the body.

Particularly, the correct formation of mammalian brain structures requires HS expression, as evidenced in the patterning defects and severe errors in midline axon guidance exhibited by mice lacking the HS-polymerizing enzyme Ext1 [50]. The disruption of the HS-modifying enzyme *N*-deacetylase and *N*-sulfotransferase 1 (Ndst1) results in severe developmental defects in the forebrain and forebrain-derived structures [51]. Additionally, the loss of enzymes responsible for the biosynthesis of the linkage region or HS chain can induce defects in bone growth and skeletal deformities [52,53,54,55,56,57], demonstrating that HS also has crucial roles in bone formation and skeletal development.

## 3. Diseases Caused by Ablation of HS Metabolism

Some human disorders are caused by mutations in genes associated with HS synthesis. Mutations in the xylosyltransferase (*XYLT1*) gene encoding an enzyme involved in the formation of the linkage region leads to Desbuquois dysplasia type 2 (Phenotype MIM number: 615777), which is characteristic of skeletal abnormalities, including pre- and postnatal growth retardation, advanced carpal ossification, and short limb bone with a prominent trochanter in the femur [10,11,58,59]. A gene mutation of *XYLT2*, the isoform of *XYLT1*, results in spondylo-ocular syndrome (MIM: 605822), which is characterized by bone fragility, cataracts, and hearing defects [60,61]. Genetic defects in the galactosyltransferase genes *B4GALT7* and *B3GALT6* are associated with Ehlers–Danlos syndrome spondylodysplastic type 1 (MIM: 130070) and spondyloepimetaphyseal dysplasia with joint laxity type 1 (MIM: 271640) or Ehlers–Danlos syndrome spondylodysplastic type 2 (MIM 615349), respectively. These disorders have overlapping features, such as short stature and osteopenia [62,63,64,65,66,67]. Multiple hereditary exostoses types 1 and 2 (MIM: 133700 and 133701) are caused by loss-of-function mutations in *EXT1* and *EXT2*, which encode proteins that form a heterooligomeric complex that catalyzes HS polymerization [68]. Multiple hereditary exostoses are autosomal dominant disorders characterized by multiple projections of bone capped by cartilage, resulting in deformed legs, forearms, and hands [69]. Defects in HS–modifying enzyme HS6ST1 can result in hypogonadotropic hypogonadism 15 with or without anosmia, which is characterized by absent sexual maturation and low circulating levels of gonadotropins and testosterone [70]. In addition, hyperglycemia in diabetes enhances HS degradation or affects the synthesis of HSPGs, consequently altering the HSPG structure and contributing to diabetic blood vessel complications, including retinopathy [71,72].

Because HS degradation is sequentially catalyzed by lysosomal enzymes, genetic defects in any of the enzymes can cause MPS (Figure 2). The details of MPSs are described in the next section.

## 4. Pathophysiological Roles of HS in MPSs

The impaired GAG degradation in MPSs is due to the deficiency or absence of lysosomal enzymes involved in the catabolism of GAGs, leading to the intracellular and extracellular accumulation of GAGs. GAG storage materials in MPSs are varied and include HS, dermatan sulfate, chondroitin sulfate, keratan sulfate, and hyaluronic acid, depending on the defective enzyme. Although the typical clinical features of patients with MPSs include skeletal and joint deformation, cardiovascular dysfunction, respiratory defects, and hearing and vision loss, neurological manifestations with intellectual or cognitive disability are observed only in particular types of MPSs where HS is accumulated [27,73]. Among the types of MPS, HS deposition is observed in patients with MPS I (MIM: 607014/607015/607016), II (MIM: 309900), IIIA (MIM: 252900), IIIB (MIM: 252920), IIIC (MIM: 252530), IIID (MIM: 252940), and VII (MIM: 253220). The causative genes are *IDUA*, *IDS*, *SGSH*, *NAGLU*, *HGSNAT*, *GNS*, and *GUSB*, respectively [28,29,30,31,32,33,34,35,36,37]. The most likely causes of the various symptoms in different MPS types are the different distribution patterns of GAG species throughout the body. The accumulation of HS but not other GAGs is found in virtually all cell types, including neuronal cells, and may be linked to the phenotypes of neuronopathic MPSs [74].

The accumulation of HS, as well as of other GAGs, directly affects lysosomal morphology, causing hypertrophy and increasing the number of lysosomes in cells. These changes also impact lysosomal function, thereby leading to abnormal membrane compositions and the secondary storage of different materials, impaired intracellular vesicle trafficking, impaired autophagy, mitochondrial dysfunction, and intracellular ionic imbalance, which causes cell function dysregulation and inflammation [75,76] (described in detail subsequently). Additionally, decreased HS degradation in the lysosomes limits the recycling of HSPGs, causes the accumulation of membrane-bound cell-surface HSPGs, and dysregulates morphogen/growth factor–receptor interactions and signal transduction [27]. Since HS and HSPGs are crucial in the development of many tissues and organs, especially in central nervous system (CNS) and musculoskeletal development, the final consequences of the pathogenic accumulation of HS caused by genetic defects affecting HS-degrading enzymes are CNS and skeletal abnormalities in patients with certain types of MPSs [26].

### 4.1. Lysosomal Dysfunction in MPSs

The lysosome is a critical organelle that contains the enzymes required for catabolizing macromolecules and processes the clearance/recycling of intra- and extracellular materials under acidic conditions through the endosomal-autophagic-lysosomal system [77]. Lysosomes are involved in several distinct pathways of degradation: macroautophagy, the most prevalent autophagic pathway for degrading intracellular components involving the fusion of autophagosomes to lysosomes [78]; endosomal degradation, which begins with the endocytosis of extracellular substances and ends in the fusion of late endosomes to lysosomes for the breakdown of the substrates [77]; microautophagy, an autophagic pathway involving lysosomal pinocytosis of cytosolic materials [79]; and chaperone-mediated autophagy, a lysosome-dependent proteolytic pathway responsible for degrading one-third of cytosolic proteins [80]. A deficiency of lysosomal enzymes in MPSs immediately leads to the accumulation of numerous autolysosomes and endolysosomes containing many partially digested macromolecules and old/damaged organelles in cells. The abovementioned autophagic and endosomal degradation pathways may be simultaneously impaired, which in turn may result in the loss of the cellular quality control system maintaining cell and organelle homeostasis.

### 4.2. Secondary Storage of Gangliosides

Gangliosides are glycosphingolipids abundant in the CNS and lipid raft domains in the plasma membrane and play important roles in cell proliferation, differentiation, adhesion and signaling, and in cell–cell communication [81]. GM2 and GM3 ganglioside levels are increased in the brains of patients with MPSs [82,83,84]. Evidence has shown that accumulated GAGs inhibit the activity of ganglioside-degrading enzymes [85,86,87,88]. The accumulation of GM2 ganglioside induces ectopic dendrite growth [89]. Ganglioside accumulation could also disrupt Ca^2+^ homeostasis and induce endoplasmic reticulum stress, resulting in neural cell apoptosis [90]. Since changes in the composition of the lipid raft can affect the functions of many ion channels and transporters, the accumulation of gangliosides is thought to be responsible, at least partly, for neural cell dysfunction and death [91]. All these neuronal responses to the abnormal accumulation of gangliosides secondary to GAG deposition are likely associated with brain pathology in MPSs.

### 4.3. Mitochondrial Dysfunction

The turnover (clearance/recycling and biogenesis) of mitochondria, as well as of other organelles, is regulated by autophagy for the maintenance of normal cellular function [92]. As discussed above, the pathological accumulation of GAGs, including HS, in MPSs impairs the autophagic–lysosomal system, leading to the impaired clearance/recycling of damaged mitochondria and subsequent mitochondrial dysfunction. Mitochondria generate ATP by oxidative phosphorylation and function in the maintenance of intracellular Ca^2+^ homeostasis, oxidative stress regulation, and lipid metabolism [93,94]. These processes are particularly important for neuronal physiology because neurons largely depend on oxidative phosphorylation for energy production and on the precise regulation of intracellular Ca^2+^ concentrations for optimal neuronal signaling [95]. Therefore, mitochondrial dysfunction is one possible mechanism leading to the neurodegeneration characteristic of certain types of MPSs [96,97].

## 5. Therapies for MPSs through the Reduction of GAGs

Therapies for MPSs include enzyme replacement therapy (ERT) and hematopoietic stem cell transplantation (HSCT). Both therapies are based on the exogenous replacement of enzymes missing in patients with MPSs. In ERT, enzymes are administered directly into circulation by intravenous (in some cases intracerebroventricular or intrathecal) injection, whereas HSCT provides enzymes through their production and release from engrafted cells. Lysosomal enzymes are incorporated into cells mediated by mannose-6-phosphate receptors to be delivered to lysosomes where they degrade the corresponding substrates [98]. ERT is currently available for MPS types I, II, IVA, VI, and VII [99] and is mostly effective for some somatic disorders. However, it cannot address CNS disorders because the blood–brain barrier (BBB) interferes with the delivery of enzymes administered intravenously to brain tissues.

**Figure 3:**
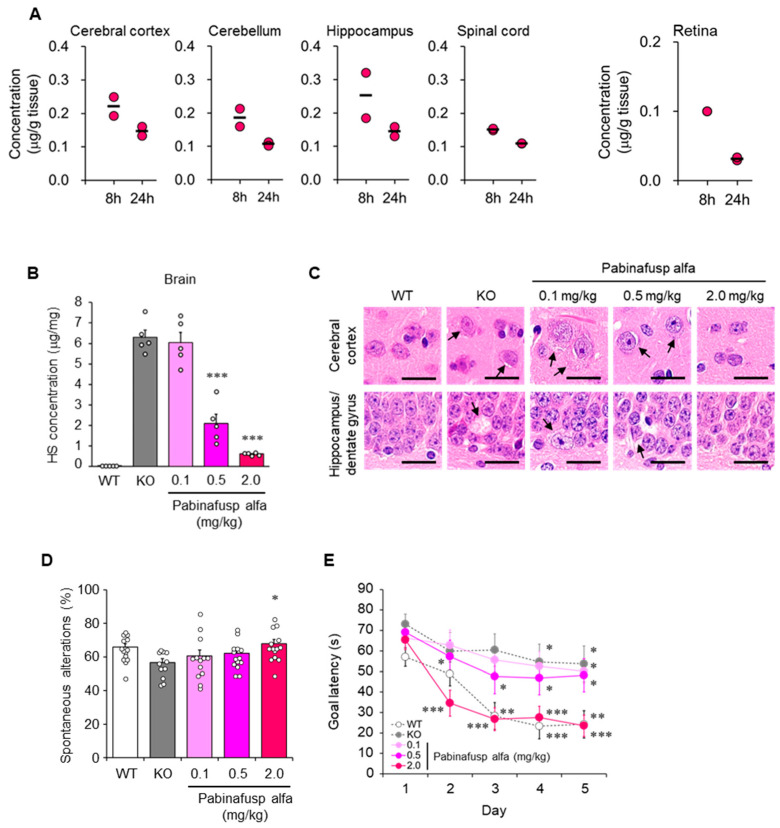
Central nervous system (CNS) distribution and efficacy of pabinafusp alfa. (**A**) CNS distribution of pabinafusp alfa in cynomolgus monkeys after a single intravenous administration at 5 mg/kg. Individual values are plotted. Bars indicate the mean. Except for retina data, the graphs were reproduced from the article by Sonoda et al. [105]. Copyright© 2018 licensed under Creative Commons Attribution License (CC BY-NC-ND 4.0). (**B**–**E**) Heparan sulfate (HS) concentrations in the brain, brain histopathology, and neurological function of mucopolysaccharidosis (MPS) II mice intravenously administered with pabinafusp alfa at indicated dosages for 36 weeks. (**B**) HS concentrations in the brain. Values are expressed as mean ± SEM (*n* = 5). *** *p* < 0.001 versus KO control (Dunnett’s test). (**C**) Brain samples stained with hematoxylin and eosin. Arrows indicate vacuolation or swelling of neuronal cells. Scale bars, 20 mm. (**D**) Rate of spontaneous alterations representing the immediate working memory assessed by Y-maze test. Values are mean ± SEM (*n* = 11–14). * *p* < 0.05 between KO and the treatment group (Dunnett’s test). (**E**) Goal latency as spatial learning ability evaluated using the Morris water maze test. Values are means ± SEM (*n* = 11–15). ** *p* < 0.01, *** *p* < 0.001 versus day 1 within each group (paired t-test). WT, wild-type mice; KO, vehicle-treated MPS II mice. Reproduced from the article by Morimoto et al. [107]. Copyright© 2022 licensed under Creative Commons Attribution License (CC BY-NC-ND 4.0).

The treatment of CNS manifestations in MPSs has been a critical but unmet need. However, a BBB-crossing technology based on the physiological mechanism of receptor-mediated transcytosis has been pioneered by Pardridge and colleagues [100,101,102,103,104] and paved the way for addressing this research gap. Recently, pabinafusp alfa, a BBB-penetrating fusion protein consisting of an anti-human transferrin receptor (TfR) antibody and IDS, was approved in Japan for the treatment of patients with all, including neuronopathic, forms of MPS II [105,106,107,108,109,110,111,112,113,114]. Nonclinical studies have shown that pabinafusp alfa is distributed in the brain parenchyma of monkeys (Figure 3A) and mice and reduces HS levels in both peripheral and CNS tissues of MPS II mice [105,106,107,108]. The clearance of HS from the brain was accompanied by reduced neurodegeneration and neurobehavioral impairments (Figure 3B–E) [106,107]. These findings suggest that HS deposition in the brain is closely associated with the pathogenesis of neurological manifestations in MPS II and that brain HS is a critical therapeutic target in neuronopathic MPS II.

**Figure 4:**
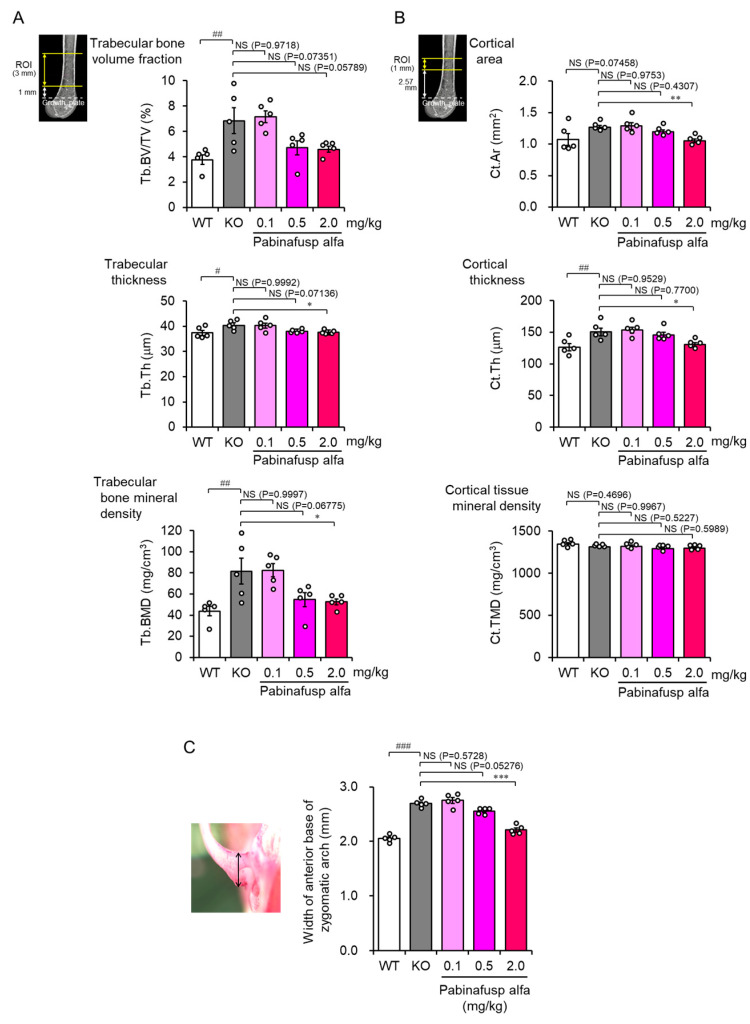
Correction of femoral and facial bone abnormalities in mucopolysaccharidosis (MPS) II mice after a 36-week treatment with pabinafusp alfa. (**A**,**B**) Three-dimensional X-ray micro-computed tomography imaging analysis was performed to measure bone morphological parameters of the trabecular (**A**) and cortical (**B**) bones of the femur. The top images present the regions of interest for the analysis. (**C**) Mice skulls were exposed after removing the scalp and immersed in 70% ethanol for a few days. After cleaning the remaining scalp, the width of the anterior base of the zygomatic arch (double-headed arrow in the photo on the left) was measured using a caliper. Bone samples obtained from a 36-week repeated dose study were analyzed [107] (our unpublished results). Values are mean ± SEM (*n* = 5). ^#^ *p* < 0.05, ^##^ *p* < 0.01, and ^###^ *p* < 0.001, WT vs. KO; * *p* < 0.05, ** *p* < 0.01, and *** *p* < 0.001, KO vs. each treatment group (Dunnett’s test). NS, not significant; WT, wild-type mice; KO, vehicle-treated MPS II mice; Tb.BV/TV, trabecular bone volume fraction; Tb.Th, trabecular thickness; Tb.BMD, trabecular bone mineral density; Cr.Ar, cortical area; Cr.Th, cortical thickness; Cr.TMD, cortical tissue mineral density.

As mentioned previously, musculoskeletal abnormalities are common features of MPSs. Patients with MPS III in which HS is the only primary accumulated substance show less severe skeletal manifestations than other types of MPSs. However, joint stiffness or contractures are frequently observed in such patients [115,116], indicating that HS deposition is involved, at least in part in the pathogenesis of musculoskeletal abnormalities in MPSs. Furthermore, ERT may have a limited effect on musculoskeletal symptoms, probably due to the low distribution of the drugs to the bone and cartilage. Nevertheless, several clinical studies have shown that early ERT initiation can prevent bone deformities to some extent in patients with MPSs [117]. Animal studies using MPS II mice have also revealed the potential efficacy of ERT in mitigating skeletal malformation (Figure 4) [118,119].

Alterations in HS synthesis or degradation are also responsible, at least partly, for retinal dysfunction in diabetes [71,72]. Retinopathy is also observed in patients with MPSs, including MPS II [120]. However, conventional ERT does not alleviate retinopathy [121], probably because the blood-retinal barrier, which structurally resembles the BBB [122], interferes with drug penetration into the retina. While pabinafusp alfa was detected in the eyes and retina of monkeys after intravenous administration (Figure 3A) [108], its efficacy against HS deposition in the retina and retinopathy in MPS II animals needs further elucidation.

## 6. HS as a Biomarker for MPSs

### 6.1. Elevation of HS Levels in Patients with MPSs

**Figure 5:**
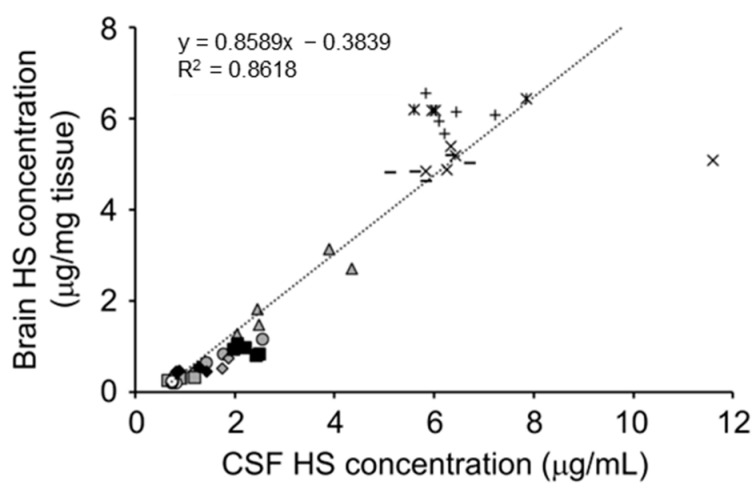
Correlations of heparan sulfate (HS) concentrations between brain and CSF. Values are derived from repeated dose studies of pabinafusp alfa in mucopolysaccharidosis (MPS) II mice [106]. The linear regression equations and correlation coefficients (R^2^) are shown in the figure. Reproduced from the article by Morimoto et al. [106]. Copyright© 2021 licensed under Creative Commons Attribution License (CC BY-NC-ND 4.0).

Since HS is the primary storage material in MPS types I, II, III, and VII [28,29,30,31,32,33,34,35,36,37], its accumulation may reasonably be used as a hallmark of these diseases. Indeed, patients with these MPS types show elevated HS levels in the blood and urine [123,124,125], which reflect the level of HS accumulation, mainly in peripheral tissue and organs. However, as the measurement of HS accumulation in the CNS, which is likely associated with brain pathology and neurological manifestations, by brain biopsy is ethically unacceptable and unpractical due to the high invasiveness and infectious risk. Animal studies using MPS II mice have provided variable information in this regard, demonstrating that HS concentrations in the cerebrospinal fluid (CSF) were closely correlated with brain HS concentrations (Figure 5) [106,126] and suggesting that CSF HS could indicate the level of HS accumulation in the brain. Clinical studies have shown that HS concentrations in the CSF of patients with MPS types I, II, and III were higher than in those without MPS [109,127,128,129,130,131,132,133], probably reflecting the elevation of HS levels in the brain of the patients.

### 6.2. HS Concentrations in the CSF and Disease Severity in MPSs

MPS I includes Hurler (severe type: MPS IH), Hurler–Scheie (intermediate type: MPS IH/S), and Scheie (attenuated type: MPS IS) syndromes and features a continuous spectrum of disease severity. Patients with MPS IH exhibit neurological, cardiovascular, and respiratory deficiencies resulting in early childhood death, whereas in MPS IS, disease progression is gradual, and patients have no neurocognitive impairment [134,135]. The genotype–phenotype relationship seems unclear, and the differences in CSF HS levels across the subtypes of MPS I have not been examined.

MPS II is divided into two types, a rapidly progressive severe type and a slowly progressive attenuated type. Patients with the severe type show normal development until 3–4 years of age when cognitive functions deteriorate, whereas patients with the attenuated type have less severe clinical symptoms and no CNS involvement in general but sometimes present with neurological symptoms at an advanced stage of the disease [30,136]. The disease severity of MPS II may depend on the type of *IDS* mutation [31]. Additionally, the mean CSF HS concentrations of patients with the severe type were higher than those of patients with the attenuated type with minimal overlapping [110,137,138]. These results suggest that levels of CSF HS levels are associated with disease severity and have a certain threshold level for distinguishing between the severe and attenuated types.

Among the four subtypes of MPS III, MPS IIIA, which is caused by *SGSH* mutations, is the most common subtype. MPS IIIA is clinically classified into a rapidly or slowly progressive form based on the diagnosis, severity, and mutation of the causative gene [135]. A prospective study reported that pediatric patients with rapidly progressive MPS IIIA reached a plateau in development by 30 months of age and regressed rapidly after 40–50 months of age, whereas children with the slowly progressive form exhibited a more prolonged course [139]. CSF HS levels in the rapidly progressive group were elevated compared with those in the slowly progressive group, although some overlapping values were noted [139], suggesting an association between CSF HS levels and disease severity in MPS IIIA.

Clearly, the pathological changes in the brains of patients with the abovementioned MPS types are initiated by HS accumulation in neural and glial cells, as indicated by CSF HS levels. However, the definitive correlation of disease severity in individual patients with CSF HS levels has yet to be fully elucidated in humans, likely because of a wide variation in patients’ background, such as age and complications, as well as the rarity of the disease. Additionally, the pathways from the initial event of HS deposition in CNS tissues to the resultant neurological symptoms are interdependent and complex, making the precise prediction of disease severity difficult if based merely on the CSF HS level at a single specific time point (also see the discussion in Section 6.4). Even in this case, however, HS deposition in the brain could be used as a biomarker of neurodegeneration resulting in CNS manifestations, which may be assessed using CSF HS levels as a surrogate biomarker.

### 6.3. Measuring Therapeutic Response Using CSF HS Concentration

HSCT is considered the primary treatment for MPS IH and can increase the life span of patients, especially at below two years of age and prior to the onset of cognitive impairment [140,141,142]. HSCT decreases HS and DS concentrations in the blood and urine [143], but CSF HS concentrations after HSCT have not been evaluated. However, post-HSCT intrathecal ERT with a recombinant iduronidase decreases CSF HS concentrations in patients with MPS IH, and the percent decrease in a certain species of HS was positively associated with the percent change in IQ score from baseline to two years, indicating a link between the treatment response of CSF HS and neurocognitive outcomes in MPS IH [132].

**Figure 6:**
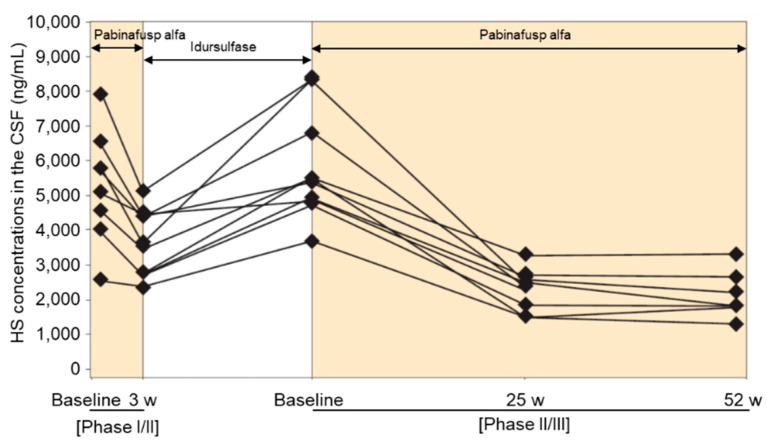
Time course of heparan sulfate (HS) concentrations in the cerebrospinal fluid (CSF) of participants in both phase I/II and phase II/III clinical trials of pabinafusp alfa. Reproduced from the article by Yamamoto and Kawashima [145] with modifications. Copyright© 2022 licensed under Creative Commons Attribution License (CC BY).

BBB-penetrating ERT for MPS II is available for clinical use in Japan and is being evaluated in clinical trials in many other countries [109,110,111,114]. Pabinafusp alfa, a BBB-penetrating and TfR-targeting antibody fusion enzyme, decreased CSF HS concentrations in patients with MPS II and exerted positive effects on neurocognitive developments in most participants (21/28) of a 52-week phase II/III clinical trial in Japan [110]. The results of a phase II study in Brazil also showed decreased CSF HS levels after treatment with pabinafusp alfa along with positive neurocognitive changes despite a short study period of 26 weeks [111]. Idursulfase, a recombinant IDS that does not cross the BBB, failed to decrease CSF HS concentrations and provided no neurocognitive benefits in patients with MPS II [144,145]. Intriguingly, CSF HS levels in the patients that participated in the clinical study increased after switching from pabinafusp alfa treatment to idursulfase treatment as standard therapy in the interim period but decreased again with the resumption of pabinafusp alfa in the next trial (Figure 6) [145]. The results demonstrate the capacity of pabinafusp alfa to reduce CSF HS concentrations via drug delivery through the BBB into the brain. Although a linear relationship between the decrease in CSF HS levels and neurocognitive improvement has not been established, the results from clinical studies nevertheless consistently confirm it.

Notably, the intrathecal administration of idursulfase to patients with MPS II or recombinant sulfamidase to patients with MPS IIIA decreased GAG/HS concentrations in the CSF but was hardly effective for cognitive function in most cases [146,147,148,149,150], casting doubt on the use of CSF HS as a surrogate biomarker for brain HS deposition and neurological dysfunction. In this regard, the biodistribution of enzymes by direct injection into the lumbar spine may well vary from that via intravenous administration of BBB-penetrating enzymes. BBB-penetrating enzymes can be delivered to most regions of the brain because the BBB exists in the capillaries throughout the brain. Conversely, while intrathecally administered enzymes may be able to enter the brain, wide distribution in the brain cannot be expected because the diffusion of large molecules through the brain parenchyma is extremely limited [151]. Therefore, as a biomarker, CSF HS should be cautiously used to evaluate the therapeutic response, considering the route of administration and distribution property of the drug. Nevertheless, since HS is a direct target of defective gene products in certain types of MPS, CSF HS may be a more reliable and sensitive biomarker for treatment response to BBB-penetrating ERT for CNS disorders than other candidate marker molecules associated with neuroinflammation and neurodegeneration.

### 6.4. Lessons Learned from Animal Studies

Results from animal studies provide valuable insight into the pathophysiological roles of HS and the use of HS measurement in MPSs. Unlike human participants, experimental inbred mouse models have uniform genetic backgrounds for which numerous siblings can be generated, thereby ensuring more robust pathological analyses and enabling the conduct of therapeutic intervention studies with minimal variation in their age, duration of disease, and disease severity. Our murine MPS II model is introduced as a typical example.

**Figure 7:**
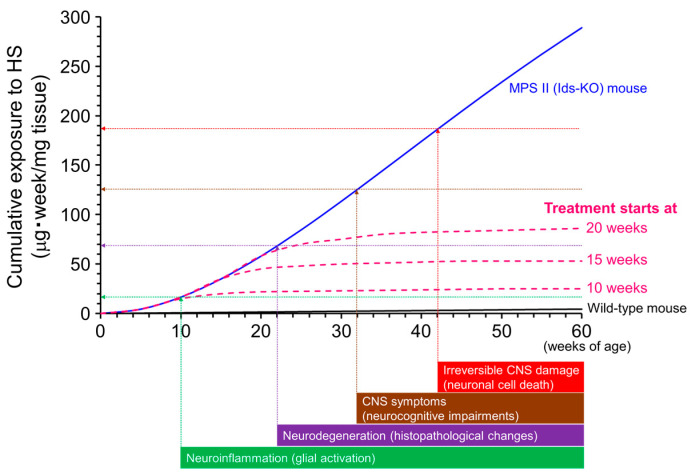
Conceptual diagram of the association between the cumulative exposure of the brain to heparan sulfate (HS) and neurological impairments. The cumulative exposure levels estimated from the actual brain HS concentrations in mucopolysaccharidosis (MPS) II mice increase with age (blue curve). Vertical arrows are the presumed times of onset of neurological events. Horizontal arrows are the postulated levels of cumulative exposure of the brain to HS when corresponding neuronal events occur. Values of the y-intercepts are tentative. The pink dashed curves indicate the presumed cumulative exposures to HS if HS-decreasing treatment for the brain was started at different weeks of age. Note that this graph is simplified to help understand the role of cumulative exposure of the brain to HS over time. Other factors associated with age may be involved in the development of central nervous system (CNS) disorders, but these are not under consideration here. Reproduced from the article by Morimoto et al. [106]. Copyright© 2021 licensed under Creative Commons Attribution License (CC BY-NC-ND 4.0).

*Ids*-KO mice recapitulate many features found in patients with MPS II, including loss of IDS enzyme activity, GAG accumulation throughout the body, skeletal deformities, walking disturbance, and neurological impairment [106,107,152]. We have established and used *Ids*-KO mice expressing human TfR (hTfR-KI/*Ids*-KO mice) as a murine model of MPS II for the pharmacological evaluation of anti-human TfR antibody fusion IDS [105]. The MPS II mice showed elevated HS/DS levels in peripheral and CNS tissues [106,107]. Brain HS concentrations in the MPS II mice increased rapidly with age but did not change substantially after 20 weeks of age. However, disease symptoms worsened progressively even after HS concentrations in the brain plateaued [106], suggesting that a temporal factor is also involved in the progression of neurological impairments. Therefore, we hypothesized that the cumulative exposure of the brain to HS is associated with neurodegeneration and cognitive abnormalities [106]. In other words, a continuous elevation in the brain HS concentration increases the risk of neural damage and neurological disorders (Figure 7). HS deposition in the brain induces neuroinflammation, which may precede neural cell damage [153,154]. A sustained exposure of glial cells and neurons to elevated HS levels could induce neurodegeneration and neurocognitive impairment and eventually result in irreversible CNS damage (neural cell death). Theoretically, however, a decrease in brain HS concentration can decrease the slope of the curve for cumulative HS exposure, which could prevent or at least slow disease progression or onset (Figure 7, pink dashed curves). The reduction in brain HS levels may also reverse neurological symptoms before irreversible damage to neuronal cells occurs. If our model is applied in clinical practice, we can speculate that early treatment can increase the chance of preventing neurological symptoms in patients with MPS II, which is a generally accepted idea. In fact, a study found that two sibling patients with MPS II who had the same *IDS* mutation showed markedly divergent developmental trajectories following ERT [138]. One sibling who was diagnosed at two years of age and subsequently received conventional ERT with idursulfase developed MPS II-related CNS signs and symptoms thereafter. The other (younger) sibling was diagnosed prenatally, and conventional ERT was started at one month of age. However, he was switched to BBB-penetrating ERT with pabinafusp alfa at one year and eleven months. The younger sibling showed normal development at three years and eleven months, along with a significant reduction in CSF HS levels [138]. This case report provides a representative example of the importance of the early initiation of CNS HS clearance therapy in patients with neuronopathic MPS II.

**Figure 8:**
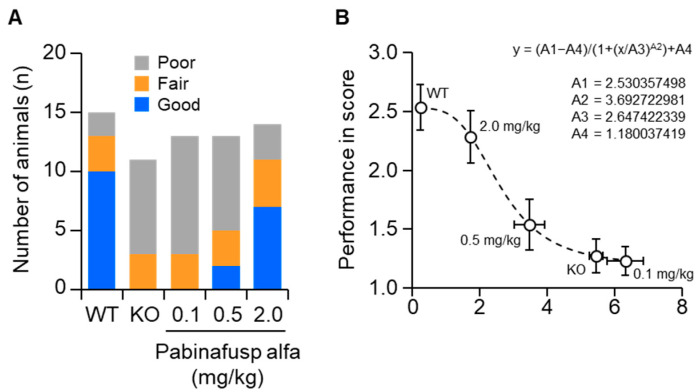
Correlation of cerebrospinal fluid (CSF) heparan sulfate (HS) levels with performance on a spatial-learning ability test. The Morris water maze test was performed in a 36-week repeated-dose study on mucopolysaccharidosis (MPS) II mice. (**A**) The performances of mice were classified as good, fair, or poor based on the goal latency response of WT and KO mice on trial day five, as shown in Figure 3. (**B**) Correlation of the performance score with CSF HS concentration. The performance in the water maze test was scored as 1 (poor), 2 (fair), or 3 (good). The curve was fitted using non-linear least squares regression, as indicated in the figure. WT, wild-type mice; KO, vehicle-treated MPS II mice. Reproduced from the article by Morimoto et al. [107]. Copyright© 2022 licensed under Creative Commons Attribution License (CC BY-NC-ND 4.0).

Results from pharmacological studies on the treatment of MPS II mice with the BBB-penetrating IDS pabinafusp alfa support the notion of an association between HS deposition in the brain and progression of neurological impairment. Treatment of MPS II mice with pabinafusp alfa decreased HS concentrations in the brain, as well as in peripheral tissues, in a linear dose-dependent manner [106,107]. A similar dose-dependent response was observed in histopathological changes in most regions of the mouse brain [107]. Additionally, improvements in performance on neurological function tests were also dose-dependent [107]. Intriguingly, performance scores for a spatial-learning ability test were correlated with CSF HS concentrations (Figure 8) [107]. These results provide nonclinical evidence that justifies the use of CSF HS as a surrogate biomarker for the early assessment of BBB-penetrating ERT response in neuronopathic MPS II. However, caution should be taken when applying it in the clinical setting (as mentioned above). This concept may be extended to other types of MPS with CNS manifestations.

## 7. Conclusions

HS, as a component of HSPGs, plays many critical roles in development and maintenance processes in the body, especially in the CNS and skeletal tissues. Maintaining the balance between the biosynthesis and degradation of HS is important. Once the balance is broken, the regulation of the development and maintenance of tissue homeostasis is disrupted, increasing the risk of disease development. Several diseases are caused by defects in molecules involved in HS biosynthesis or degradation. Of these, MPSs are inherited lysosomal storage disorders in which enzymes for GAG degradation are deficient or inactive. The pathological accumulation of HS is observed in some types of MPSs and leads to neurological manifestations with cognitive disability in severe cases. Animal studies have shown that HS deposition in the brain causes neurodegeneration and neurological manifestations through multiple mechanisms and that CSF HS concentrations are significantly correlated with those in the brain. Additionally, CSF HS levels after BBB-penetrating ERT could predict improved neurological function in MPS II mice. Although clinical evidence suggests that CSF HS levels are also associated with disease severity in humans, an argument against this notion has been proposed based on the finding that CSF HS levels do not increase despite disease progression. This discrepancy may be due to the underestimation of the temporal factor in HS exposure. Our hypothesis on the relationship between the cumulative HS exposure level in the brain and brain pathology and neurological symptoms can explain this discrepancy. Disease onset and progression may be closely tied to the cumulative HS exposure levels in the brain; therefore, a substantial amount of time is needed for CNS disorders to manifest from the accumulation of substrates (Figure 7). Conversely, considerable time is required to produce beneficial clinical outcomes from the reduction of HS levels in the CNS through treatment with BBB-penetrating ERT. Although the correlation between CSF HS concentrations and CNS symptoms needs to be conclusively validated in long-term clinical studies, CSF HS is a potential biomarker for the early assessment of therapeutic response in CNS diseases associated with MPSs.

## Data Availability

All data supporting the conclusion of this study are available within the article.
